# Vaccination with Ad5 Vectors Expands Ad5-Specific CD8^+^ T Cells without Altering Memory Phenotype or Functionality

**DOI:** 10.1371/journal.pone.0014385

**Published:** 2010-12-22

**Authors:** Natalie A. Hutnick, Diane G. Carnathan, Sheri A. Dubey, Kara S. Cox, Lisa Kierstead, George Makadonas, Sarah J. Ratcliffe, Marcio O. Lasaro, Michael N. Robertson, Danilo R. Casimiro, Hildegund C. J. Ertl, Michael R. Betts

**Affiliations:** 1 Department of Microbiology and Center for AIDS Research, University of Pennsylvania School of Medicine, Philadelphia, Pennsylvania, United States of America; 2 Merck Research Laboratories, Vaccine Basic Research, West Point, Pennsylvania, United States of America; 3 Department of Biostatistics and Epidemiology, University of Pennsylvania School of Medicine, Philadelphia, Pennsylvania, United States of America; 4 The Wistar Institute, Philadelphia, Pennsylvania, United States of America; Statens Serum Institute, Denmark

## Abstract

**Background:**

Adenoviral (Ad) vaccine vectors represent both a vehicle to present a novel antigen to the immune system as well as restimulation of immune responses against the Ad vector itself. To what degree Ad-specific CD8^+^ T cells are restimulated by Ad vector vaccination is unclear, although such knowledge would be important as vector-specific CD8^+^ T cell expansion could potentially further limit Ad vaccine efficacy beyond Ad-specific neutralizing antibody alone.

**Methodology/Principal Findings:**

Here we addressed this issue by measuring human Adenovirus serotype 5 (Ad5)-specific CD8^+^ T cells in recipients of the Merck Ad5 HIV-1 vaccine vector before, during, and after vaccination by multicolor flow cytometry. Ad5-specific CD8^+^ T-cells were detectable in 95% of subjects prior to vaccination, and displayed primarily an effector-type functional profile and phenotype. Peripheral blood Ad5-specific CD8^+^ T-cell numbers expanded after Ad5-HIV vaccination in all subjects, but differential expansion kinetics were noted in some baseline Ad5-neutralizing antibody (Ad5 nAb) seronegative subjects compared to baseline Ad5 nAb seropositive subjects. However, in neither group did vaccination alter polyfunctionality, mucosal targeting marker expression, or memory phenotype of Ad5-specific CD8^+^ T-cells.

**Conclusions:**

These data indicate that repeat Ad5-vector administration in humans expands Ad5-specific CD8^+^ T-cells without overtly affecting their functional capacity or phenotypic properties. This is a secondary analysis of samples collected during the 016 trial. Results of the Merck 016 trial safety and immunogenicity have been previously published in the journal of clinical infectious diseases [1].

**Trial Registration:**

ClinicalTrials.gov NCT00849680 [NCT00849680]

## Introduction

Vectors based on the human Adenovirus serotype 5 (Ad5) are currently leading candidates for vaccines designed to elicit cellular immunity. Studies both in animals and humans have demonstrated that Ad5-vectors are capable of inducing potent and sustained transgene product specific CD4^+^ and CD8^+^ T-cell responses [2], [3], [4]. Additionally, these vectors have been generally safe and well tolerated [1], [5], [6]. However, one major hurdle to Ad-vector based vaccines is the presence of pre-existing Ad-specific immunity.

Most studies of pre-existing Ad-specific immunity have focused on neutralizing antibodies (nAb). In animals and humans, Ad5 vaccination is less effective if there are pre-existing Ad5-specific nAbs [4], [7]. Similarly, pre-exposure to Ad5 vector reduces the efficacy of subsequent booster vaccinations, thereby limiting the ability for homologous vector boosting [8]. The prevalence of nAbs to Ad5 varies worldwide, with up to 50% of adults in the United States and as many as 90% of adults in Africa testing seropositive [9]. To overcome this limitation, rare Ad serotypes with low seroprevalence have been developed as vaccine vectors [10], [11], [12].

Ad-specific CD4^+^ and CD8^+^ T-cell responses have also been detected in humans [13], [14]. Previous studies following vaccination have found Ad5-specific CD8^+^ T-cell responses in greater than 80% including baseline seronegative subjects [15], [16]. However, their functional properties, and phenotypic characterization of Ad5-specific CD8^+^ T-cell directly *ex vivo* before and after vaccination are not well described. We have previously done an extensive characterization of Ad5-specific CD8^+^ T-cells following natural infection, however, it is unclear whether Ad-specific T-cells stimulated by vaccination are similar to those induced by natural infection [17]. Moreover, the effect of repeat homologous E1-deleted Ad5 vector administration upon pre-existing Ad-specific CD8^+^ T cells has not been assessed in human vaccine recipients.

To assess the effect of Ad vector administration on the Ad-specific CD8^+^ T-cell response in humans, we analyzed previously collected peripheral blood mononuclear cells (PBMCs) from a small subset of subjects that had been enrolled in a Phase 1 Ad5 vector human vaccine trial. This study was a basic immunological investigation designed after the completion of the original trial and a continuation of work previously performed to characterize Ad5-specific CD4^+^ T-cell responses [18], [19]. Using a whole Ad5 vector stimulation together with polyfunctional flow cytometry, we defined the prevalence, magnitude, functionality and phenotype of Ad5-specific CD8^+^ T-cells before and after Ad5-vector administration. Our results demonstrate that while Ad5-specific CD8^+^ T-cells are present in most humans and transiently expand after vaccination, they do not change in either phenotype or function.

## Materials and Methods

### Ethics Statement

IRB approval was obtained by Merck at each subject study site: Emory University, Lehigh University, Western, The Miriam Hospital Office of Research and AIDS research alliance. Written informed consent was obtained from all participants. All authors of this study were blinded to patient identifiers.

### Subjects

Frozen peripheral blood mononuclear cells (PBMCs) were obtained from unvaccinated subjects at week 0 baseline (*n* = 25), Ad5 seronegative subjects receiving one dose of 3×10^10^ vp Mrk Ad5 gag/pol/nef at week 0 and followed through week 4 (*n* = 5), Ad5 seronegative subjects receiving three doses 3×10^10^ vp Mrk Ad5 gag/pol/nef at weeks 0, 4 and 26 (*n* = 5, Ad5 neutralizing antibody titer ≤18) and Ad5 seropositive (*n* = 5, Ad5 neutralizing antibody titer >18) subjects receiving three doses 3×10^10^ vp Mrk Ad5 gag/pol/nef at weeks 0, 4 and 26 (*n* = 5) as part of the Merck phase I 016 trial (Figure S1). The vaccination dose, product, and schedule was identical to that used in the phase II Merck STEP HIV-1 vaccine trial. PBMCs were obtained from study weeks 0 (baseline), 4, 8, 18, 26, 30, 42, 52 and 78. Results of the Merck 016 trial safety and immunogenicity have been previously published in the journal of clinical infectious diseases [1]. The clinical trial registry number is NCT00849680. This is a secondary analysis of samples collected during the 016 trial.

### Vector and virus

Replication defective Adenovirus 5 (Ad5) and chimpanzee derived Ad vector of serotype 7 (AdC7) vector as well as wild-type Ad5 virus were prepared using previously described methods [20]. Vectors and virus were grown on HEK293 cells [21] in DMEM supplemented with 10% fetal calf serum, antibiotics and glutamine. Vectors and virus were purified by CsCl gradients and quality controlled. Quality controls of vectors included testing for replication-defective adenovirus, which could not be detected in the E1-deleted Ad5 vector preparations.

### Detection of Ad5 hexon transcripts by real-time PCR

CHO-CAR cells were infected at 1000 vp/cell ratio with either replication-effective Ad5 (Ad5 wt) or replication-defective Ad5rab.gp or AdC7rab.gp vectors. Non-infected cells were used as negative control. Forty-eight hours later, total RNA was isolated using TRI reagent (Sigma, MI), tested by PCR for DNA contamination, and then reversed transcribed using M-MLV Reverse Transcriptase (Invitrogen, CA). Samples were adjusted to equal amounts of gapdH (4.5×10^7^ copies) as described [22], and then Ad5 hexon sequence was amplified by PCR (initial cycle of 94°C for 5 min, 35 cycles of 94°C for 60 s, 55°C for 60 s, and 72°C for 60 s, and final cycle of 72°C for 7min) using the following primers: forward 5′ ATCATGCAGCTGGGAGAGTC, and reversee 5′ ACACCTCCCAGTGGAAAGCA. The amplicon (0.2µl) from the first PCR product was used as template for a nested quantitative real-time PCR (45 cycles at 95°C for 5 s, 54°C for 10 s, and 72°C for 15 s; acquisition was at 83°C) conducted using the LightCycler-RNA amplification kit SYBR Green 1 (Roche, IN). The following primers were used for the nested PCR: forward 5′ GACTCCTAAAGTGGTATTGT, and reverse 5′ ACACCTCCCAGTGGAAAGCA. Specificity of amplicons was confirmed by analyses of melting temperature curves and gel electrophoresis. Results are shown as the number of mRNA copies per 10^6^ copies of gapdH mRNA.

### Antibodies

Directly conjugated antibodies were obtained from the following: BD Biosciences: TNFα (Pe-Cy7), IFN-γ (Alexa700); Caltag: CD14 (APC-Alexa750), CD19 (APC-Alexa750), Ki67 (Fitc), and CD4 (Pe-Cy5.5); Beckman Coulter: CD8 (ECD), CD27 (Pe-Cy5); and R&D systems: MIP1α (FITC), and IL-2 (APC). We conjugated the following antibodies in our laboratory: CD3 (QD585), CD57 (QD565), CD45RO (QD705), and Perforin (Pacific Blue) using standard protocol supplied by the manufacturer. We obtained the unconjugated CD45RO and CD57 from AbD Serotec, perforin BD-49 from Diaclone and CD3 OKT3 from American Type Culture Collection. We obtained Pacific Blue and Quantum Dots from Invitrogen.

### Cell Stimulation and Staining

T cell responses were measured to E1-deleted Ad5 vector that expressed the rabies virus glycoprotein [23]. 2×10^6^ PBMCs were incubated overnight with 1×10^11^ Ad5 viral particles (vp) and costimulatory antibodies (αCD28/49d, 1 µg/ml each; BD Biosciences) at 37°C and 5% CO_2_ in 1 ml complete RPMI media (RMPI 1640 with 10% heat inactivated FBS, 100 U mL/ml Penicillin, 100 µg/ml streptomycin sulfate and 1.7 mM sodium glutamate)_._ A positive control was stimulated with *Staphylococcus* enterotoxin B (SEB, 1 mg/ml; Sigma-Aldrich) and a negative control received only co-stimulatory antibodies. The following morning we added monensin (Golgi Stop, 0.7 µg/ml; BD Biosciences) and Brefeldin A (1 µg/ml; Sigma-Aldrich) to each sample and incubated the cells for six hours at 37°C and 5% CO_2_. Samples were then washed in PBS and stained for viability (Aqua live/dead amine reactive dye; Invitrogen) followed by treatment with surface antibodies. The cells were permeabilized and fixed using the Cytofix/Cytoperm kit (BD Biosciences) then stained with intracellular fluorochome-labeled antibodies. Following staining cells were washed, fixed (2% paraformaldehyde in PBS) and stored at 4°C in the dark until analysis.

### Flow Cytometry

Cells were analyzed on a modified LSR II flow cytometer (BD Immunocytometry Systems) with 200,000 to 1,000,000 events collected per sample. Data was analyzed using FlowJo 8.7.1 (TreeStar). Cells were initially gated to remove doublets, followed by a lymphocytes gate on forward scatter area versus side scatter area. Dead cells were removed by gating CD3 versus Aqua blue and removing events that were Aqua blue bright. CD14^+^ and CD19^+^ cells were also removed before gating sequentially on CD3^+^, CD8^+^/CD4^+^ and CD4^−^/CD8^−^ versus IFN-γ. A gate was then made for each respective function and the Boolean gating platform used to create the array of 32 possible functional combinations. Data are reported after background subtraction of the no stimulation condition. A response greater than 0.05% after background subtraction was considered positive.

### nAb titers

Adenovirus 5 neutralizing antibody titers were measured as previously described [24]. Briefly, 2×10^4^ HEK293 cells per well in a 96 well plate were seeded for 2 days. Ad-SEAP was incubated for 1 hour at 37°C either alone or with serial dilutions of serum then added to the 95–100% confluent 293 cells and incubated for 1 hr at 37°C. Supernatant was then removed and replaced with 10% FBS in DMEM. DEAP expression as measured 24±2 hrs later with the chemiluminescent substrate from the Phospha-Light™ kit. (Applied Biosystems).

### Statistics

Linear mixed effects models were performed to test for group differences over time as well as comparisons between baseline and subsequent time points within each group. Time was considered to be a discrete variable, lessening the power of these tests compared to tests where time is a continuous variable. Spearman correlations were used to test the relationship between Ad5 nAb titers and T-cell functions at baseline. Correlations over the entire time period were computed using partial correlation coefficients controlling for individual subject effects in the repeated measurements. All data was transformed using base e. Analyses was performed using SAS 9.1.

## Results

**Figure 1:**
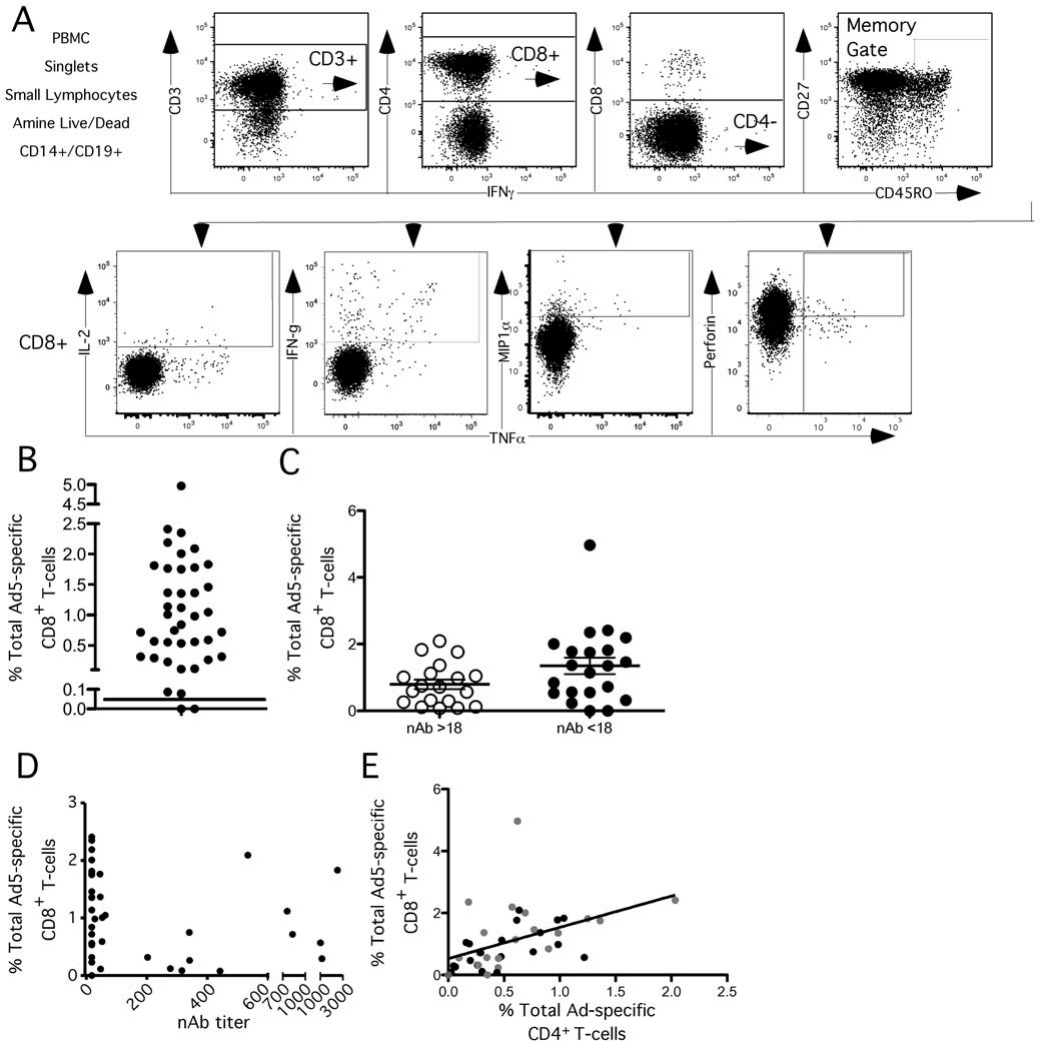
Baseline CD8^+^ T-cell responses. Forty total subjects with a range of Ad5 nAb titers were analyzed. Five seronegative (Ad5 nAb titer ≤18, gray symbols) and five seropositive subjects (Ad5 nAb titer >18, black symbols and white boxes) received Merck Ad5 gag/pol/nef as described in Methods. Each circle represents a subject with lines representing the mean. CD8^+^ T-cell responses were measured by flow cytometry following whole Ad5 vector stimulation. **A**) Gating strategy for measuring Ad5-specific T cells by intracellular cytokine staining. At least 100,000 PBMCs were collected on a modified LSR II. Singlets were selected with a FSC-A and FSC-H, followed by a lymphocytes gate, dead cell exclusion, and exclusion of contaminating CD14^+^ monocytes and CD19^+^ B-cells. CD3^+^ T-cells were selected and then CD8^+^ cells by CD8^+^CD4^−^. Central memory, effector memory and effector CD8^+^ T cells were selected before gating on each cytokine. Because cells can store perforin and these appear perforin^+^, Ad5-specific CD8^+^perforin^+^ T cells must also be positive for another function to be considered as a responding event. **B**) Total Ad-specific CD8^+^ response. The total Ad-specific CD8^+^ response was computed by summing cells making at least IL-2, IFN-γ, MIP1α, or TNFα as measured by flow cytometry. **C**) There was no difference in the magnitude of the Ad-specific CD8^+^ T-cell response between serogroups at baseline. **D**) There was no correlation between the magnitude of Ad-specific CD8^+^ T-cell responses and nAb titer at baseline. **E**) There is a correlation between the magnitude of Ad-specific CD4^+^ and Ad-specific CD8^+^ T-cell responses at baseline.

To assess the total magnitude, functionality, and phenotypes of Ad5-specific CD8^+^ T-cells, we stimulated human PBMCs with Ad5 vector and measured CD8^+^ T-cell responses by polychromatic flow cytometry (Fig. 1A). We detected Ad5-specific CD8^+^ T-cell responses in 95% of all donors at the week 0 baseline (Fig. 1B). There was no difference in frequencies of Ad5-specific CD8^+^ T-cells between baseline seropositive and seronegative subjects (Fig. 1C, p>0.05). Likewise there was no correlation between frequencies of Ad5-specific CD8^+^ T-cells and the Ad5 nAb titer at baseline (Fig. 1D, p>0.05). There was however a positive correlation between frequencies of baseline Ad5-specific CD4^+^ and CD8^+^ T-cells (Fig. 1E).

### Vector induced CD8^+^ T-cell expansion

**Figure 2:**
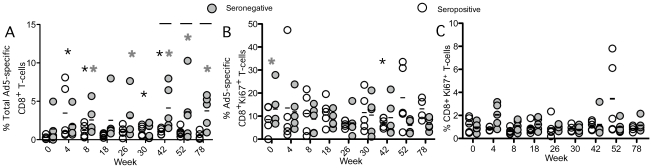
Ad-specific CD8^+^ T-cells magnitude following vaccination. Five seronegative (Ad5 nAb titer ≤18, grey circles) and five seropositive subjects (Ad5 nAb titer >18, white circles) received Merck Ad5 gag/pol/nef as described in Methods. CD8^+^ T-cell responses were measured by flow cytometry following whole Ad5 vector stimulation. Black lines recommend the mean. Grey asterics represent a significant increase from baseline in seronegative subjects and black asterics represent a significant difference from baseline in seropositive subjects. Black bars represent a significant difference between the serogroups at that time point. **A**) Percentage of Ad-specific CD8^+^ T-cells. Seronegative subjects were significantly increased above baseline at week 8 (p<0.03), 26 (p<0.03), 42 (p<0.001), 52 (p<0.01), and 78 (p<0.001). Total Ad-specific CD8^+^ T-cells were increased above baseline in seropositive subjects at week 4 (p<0.0001), 8 (p<0.03), 30 (p<0.02), and 42 (p<0.04). Serogroups significantly differed at week 42 (p<0.02), 52 (p<0.01), and 78 (p<0.003). **B**) Percentage of Ad-specific Ki67^+^ CD8^+^ T-cells. **C**) Percentage of Ki67^+^ CD8^+^ T-cells. There was a significant increase above baseline in seronegative subjects at week 4 (p<0.01) and serpositive subjects at week 52 (p<0.12).

We next examined the effect of Ad5 vector administration on the pre-existing Ad5-specific CD8^+^ T-cell response. After the initial vaccine administration, frequencies of Ad5-specific CD8^+^ T-cells in blood increased significantly above pre-vaccination frequencies in baseline Ad5 seropositive (p<0.0001) but not in baseline seronegative subjects as a group (Fig. 2A). On an individual basis, frequencies of Ad5-specific CD8^+^ T-cells increased in three of five seronegative subjects following the initial dose (data not shown). The remaining two seronegative subjects without Ad5-specific CD8^+^ T-cell expansion had large baseline responses.

Four weeks after the first homologous Ad5 vector boost at week 4, Ad5-specific CD8^+^ T-cell frequencies were higher than baseline in both subject groups (p<0.03). Following this first boost, Ad5-specific CD8^+^ T-cell responses returned to pre-vaccination levels in baseline Ad5 seropositive subjects, and were only briefly expanded again at weeks 30 and 42 following the 2nd boost at week 26 (p<0.04). In contrast, Ad5-specific CD8^+^ T-cell responses remained elevated above baseline in the seronegative cohort (p<0.03). The only time we observed a difference in Ad5-specific CD8^+^ T-cell expansion between the seronegative and seropositive groups was following the third vaccination (weeks 42, 52 and 78; p<0.03)(Fig. 2A).

The observed increase in Ad5-specific CD8^+^ T-cell frequencies was not reflected by an increase in Ki67 on Ad5-specific CD8^+^ T-cells after either the primary vaccination or subsequent boosts in either serogroup (Fig. 2B, p = N.S.). Furthermore, we detected only transient differences in global Ki67 levels on total CD8^+^ T-cells (Fig. 2C). Thus, while increases in Ad5-specific CD8^+^ T-cell frequencies were observed in both baseline Ad5 seronegative and seropositive subjects following vaccination, sustained changes or global effects on the proliferative capacity of CD8^+^ T-cells were not found.

### Transcription of late genes by E1-deleted Ad5 vectors

**Figure 3:**
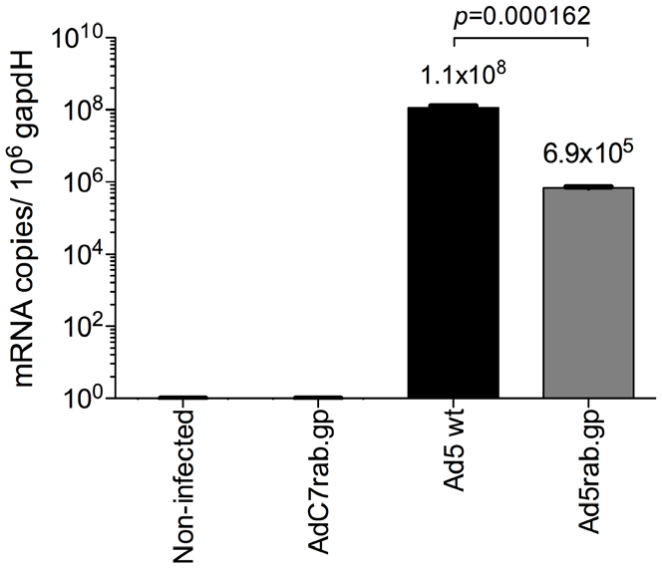
AdHu5 hexon quantitative real-time PCR. RNA isolated from non-infected cells (dashed bar) and from cells infected with AdC7rab.gp (white bar), Ad5 wt (black bar) or Ad5rab.gp (gray bar) were reverse transcribed and normalized to equal amount of gapdH mRNA copies. Ad5 hexon mRNA copies were quantified by real-time PCR using specific primers. No Ad5 hexon specific mRNA was detected in non-infected cells or cells infected with AdC7rab.gp. Ad5 hexon mRNA levels were assessed in triplicates. Averages (Ad5 wt and Ad5rab.gp) and p values from two-tail student's t test are shown on top of the bars.

E1-deleted Ad5 vectors are replication-defective as E1 gene products are needed to initiate transcription of the other viral genes. Nevertheless, cellular factors can in part compensate for loss of E1 and E1-deleted Ad vectors can thus be transcribed and even proliferate in certain cell types [25], [26]. To test if the E1-deleted Ad5 vector were able to transcribe structural late genes, and thus be recognized by CD8^+^ T cells, CHO cells stably transfected with a vector expressing the coxsackie adenovirus receptor (CAR) were infected with 1000 vp per cell of an E1-deleted Ad5 vector, 1000 vp/cell of wild-type Ad5 virus as a positive control or 1000 vp of an E1-deleted chimpanzee-derived Ad vector (AdC7) as a negative control. RNA was isolated 48 hours later and the amount of mRNA encoding the viral hexon was determined using a quantitative real-time PCR with primers designed to distinguish hexon transcripts of AdHu5 from those of AdC7. Results clearly showed that hexon was being transcribed although at levels that were at least 500 fold below those achieved by wild-type virus (Figure 3). A reduction in vector dose resulted in reduced levels of transcripts (data not shown). Cells infected with the AdC7 vector or non-infected cells were negative for Ad5 hexon transcripts.

### Vector-induced changes in Ad5-specific CD8^+^ T-cell functionality

**Figure 4:**
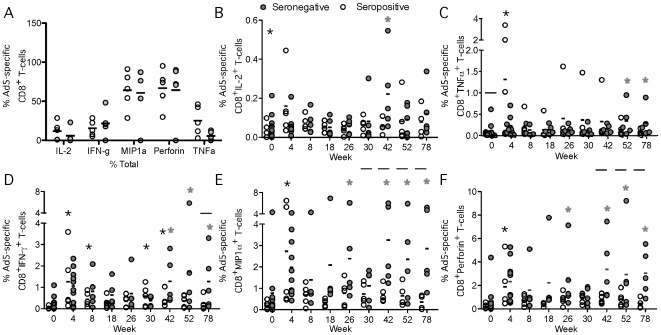
Ad specific T-cell functionality following vaccination. Ten seronegative (Ad5 nAb titer ≤18, five weeks 0–4, five weeks 0–78, grey circles) and five seropositive subjects (Ad5 nAb titer >18, white circles) received Merck Ad5 gag/pol/nef as described in Methods. Black lines recommend the mean. Grey asterics represent a significant increase from baseline in seronegative subjects and black asterics represent a significant difference from baseline in seropositive subjects. Black bars represent a significant difference between the serogroups at that time point **A**) Percent of the total Ad-specific response producing each cytokine. The total Ad-specific CD8^+^ response was computed by summing cells making at least IL-2, IFN-γ, MIP1α, or TNFα as measured by flow cytometry. The percentage of the total Ad-specific response was then computed for each cytokine. The percentage of the total response consisting of TNFα was significantly higher in seropositive subjects (p<0.0005) at baseline. **B**) Percentage of IL-2^+^ Ad-specific CD8^+^ T-cells. There was a significant increase above baseline in seronegative subjects at week 42 (p<0.01) and seropositive subjects at week 4(p = 0.015). **C**) Percentgae of TNFα^+^ Ad-specific CD8^+^ T-cells. There was a significant increase above baseline in seropositive subjects at week 52 (p<0.01) and 78 (p<0.02) and serpositive subjects at week 4 (p<0.01). There was a significant difference at baseline in the percentage of TNFα^+^ CD8^+^ T-cells between serogroups (p<0.05). **D**) Percentage of IFN-γ^+^ Ad-specific CD8^+^ T-cells. The percentage of IFN-γ^+^ CD8^+^T-cells was significantly increased above baseline in seronegative subjects at weeks 42 (p<0.004), 52 (p<0.003), and 78 (0.009) and in seropositives at weeks (4 (p<0.0001), 8 (p<0.04), 30 (p<0.01) and 42 (p<0.04). There was a significant difference in the percentage of CD8^+^IFN-γ^+^ CD8^+^ T-cells at week 78 between the serogroups (p<0.05). **E**) Percentage of MIP1α^+^ Ad-specific CD8^+^ T-cells. Seronegative subjects had a significantly increased percentage of MIP1α^+^ CD8^+^ T-cells above baseline at weeks 26 (p<0.03), 42 (p<0.001), 52 (p<0.05) and 78 (p<0.004). Seropositive subjects had a significantly increased percentage of MIP1α^+^ CD8^+^ T-cells at week 4 (p<0.0005) compared with baseline There was a significant difference in the percentage of MIP1α^+^CD8^+^ T-cells between the serogroups at weeks 30 (p<0.04), 42 (p<0.012), 52 (p<0.005), and 78 (p<0.001). **F**) Percentage of perforin^+^ Ad-specific CD8^+^ T-cells. The percentage of perforin^+^CD8^+^ T-cells was significantly increased above baseline at weeks 26 (p<0.02), 42 (p<0.001) 52 (p<0.05) and 78 (p<0.004) in seronegatives and week 4 (p<0.0005) in seropositive subject. There was a significant difference in the percentage of Perforin CD8^+^ T-cells at weeks 42 (p<0.04), 52 (p<0.02) and 78 (p<0.006).

At baseline, the majority of Ad5-specific CD8^+^ T-cells produced predominantly the effector functions MIP1α and perforin in both seropositive and seronegative subjects (Fig. 4A). The percentage of Ad5-specific CD8^+^ T-cells producing TNFα was significantly higher in baseline seropositive subjects prior to vaccination (p<0.001) but there were no differences between the groups for IL-2, IFN-γ, MIP1α or perforin.

Having observed increases in the total percentage of Ad5-specific CD8^+^ T-cells following vaccination we next determined whether vaccination affected the functionality of these cells. Consistent with the increase in Ad5-specific CD8^+^ T-cell frequencies following the initial vector vaccination, baseline seropositive subjects showed a transient increase in percentage of Ad5-specific CD8^+^ T-cells producing IL-2 (Fig. 4B, p = 0.015), TNFα (Fig. 4C, p<0.01), and IFN-γ (Fig. 4D, p<0.001) as well as the MIP1α (Fig. 4E, p<0.0005), and perforin (Fig. 4F, p<0.0002).

The functionality of Ad5-specific CD8^+^ T-cells was similar in baseline seronegative and seropositive subjects, however in baseline seronegative subjects the expansion of Ad-specific CD8^+^ T cells was delayed. Following the third vector dose baseline seronegative subjects had transiently elevated IL-2 producing CD8^+^ T-cells at week 42(Fig. 4B) and TNFα from weeks 52–78(Fig. 4C). CD8^+^ T cells that produced the effector functions IFN-γ, MIP1α and perforin were expanded for a more prolonged period of time (weeks 42–78) following the third vector dose. This delayed expansion resulted in a higher percentage of Ad5-specific CD8^+^ T-cells in baseline seronegative subjects compared with baseline seropositive subjects following the third vector dose with MIP1α significantly elevated at weeks 30–78 and perforin at weeks 42–78.

**Figure 5:**
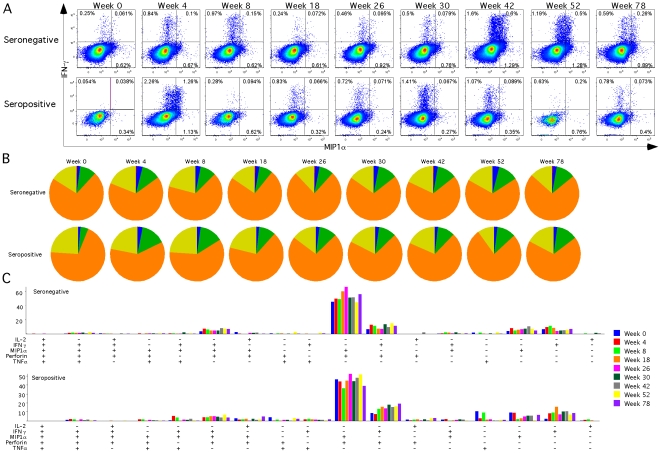
Polyfunctional Ad-specific CD8^+^ T-cell Responses. Five seronegative (Ad5 nAb titer ≤18, grey circles) and five seropositive subjects (Ad5 nAb titer >18, white circles) received Merck Ad5 gag/pol/nef as described in Methods. CD8^+^ T-cell responses were measured by flow cytometry following whole Ad5 vector stimulation. **A**) Percentage of CD8^+^ T-cells expressing IFN-γ and MIP1α. **B**) Percentage of Ad-specific CD8^+^ T-cells producing a all five (red) functions: IL-2, MIP1α, TNFα, IFN-γ and Perforin, four (blue), three (green), two (orange), or one (yellow) of the five functions at each time point. Pies represent an average of the two groups. **C**) Bars represent the percentage of Ad-specific CD8^+^ T-cells making a combination of IL-2, TNFα, MIP1α, Perforin and IFN-γ at each week. Positive symbols represent cells staining positive for a function, and minus symbols represent cells staining negative for a function.

Although we observed increases in the frequencies of Ad5-specific CD8^+^ T-cells producing various functions, the overall polyfunctionality of Ad5-specific CD8^+^ T-cells remained similar to baseline after vaccination (Fig. 5A, B) in both groups. Furthermore, there was no substantial difference between the groups in the functional combinations produced (Fig. 5C). In both groups the major response consisted of cells producing MIP1α with perforin and MIP1α with IFN-γ.

### Ad5-specific CD8^+^ Phenotype

**Figure 6:**
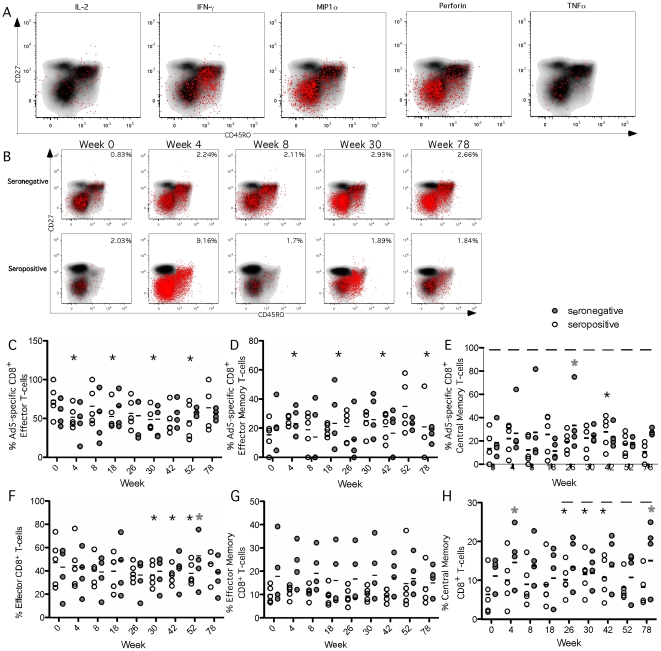
Phenotype of Ad5-specific CD8^+^ T-cells. Five seronegative (Ad5 nAb titer ≤18, gray circles) and five seropositive subjects (Ad5 nAb titer >18, white circles) received Merck Ad5 gag/pol/nef as described in Methods. CD8^+^ T-cell responses were measured by flow cytometry following whole Ad5 vector stimulation. Black lines represent the mean. Grey asterics represent a significant increase from baseline in seronegative subjects and black asterics represent a significant difference from baseline in seropositive subjects. Black bars represent a significant difference between the serogroups at that time point. **A**) The phenotype of Ad-specific CD8^+^ T-cells in a represenative donor. Black areas represent total CD8^+^ T-cells and red dots represent Ad-specific CD8^+^ T-cells expressing IL-2, MIP1α, Perforin or TNFα. **B**) Phenotype of Ad-specific CD8^+^ T-cells following vaccination. Black areas represent total CD8^+^ T-cells and red dots represent total Ad-specific CD8^+^ T-cells expressing IL-2, MIP1α, Perforin or TNFα. Numbers represent the total percetage of Ad-specific CD8^+^ T-cells. **C**) Percentage of Ad-specific CD8^+^ T-cells with an effector phenotype (CD27-CD45RO−). Seropositive subjects were significantly decreased from baseline at weeks 4 (p<0.03), 18 (p<0.04), 30 (p<0.01), 42 (p<0.009), 52 (p<0.003). **D**) Percentage of Ad-specific CD8^+^ T-cells with an effector memory phenotype (CD27-CD45RO^+^). Seropositive subjects were significantly increased at weeks 4 (p<0.05), 30 (p<0.02), 52 (p<0.001), 78 (p<0.05). **E**) Percentage of Ad-specific CD8^+^ T-cells with an central memory like phenotype (CD27^+^CD45RO^+^). Sernegative subjects were significantly increased above baseline at week 26 (p<0.02) and seropositive subjects were significantly increased from baseline at week 42 (p<0.03). There was a significant difference between the groups at all time points. (P<0.05). **F**) Percentage of total CD8^+^ T-cells with an effector phenotype (CD27-CD45RO−). Seropositive subjects were significantly decreased from baseline at weeks 30 (p<0.02), 42 (p<0.002) and 52 (p<0.04). Seronegative subjects were also significantly decreased from baseline at week 52 (p<0.01). **G**) Percentage of total CD8^+^ T-cells with an effector memory phenotype (CD27-CD45RO^+^). **H**) Percentage of total CD8^+^ T-cells with a central memory like phenotype (CD27^+^CD45RO^+^). The percentage was significantly increased in seropositive subjects at weeks 26 (p<0.02), 30 (p<0.002) and 42 (p<0.008) and seronegative subjects at weeks 4 (p<0.04), and 78 (p<0.02). There was a significant difference between the two groups at weeks 26 (p<0.05), 30 (p<0.04), 42 (p<0.03), 52 (p<0.02), and 78 (p<0.01).

To investigate whether the effector-like functionality of Ad5-specific CD8^+^ T-cells corresponded to an effector phenotype, we assessed CD45RO and CD27 expression on Ad5-specific CD8^+^ T-cells. Ad5-specific cells that produced MIP1α, and perforin were primarily of an effector-like phenotype (CD27-CD45RO-) (Fig. 6A), whereas more diverse memory subsets produced IFN-γ, TNFα, and IL-2. Approximately half of all Ad5-specific CD8^+^ T-cells had an effector like phenotype at baseline in both seronegative and seropositive subjects (Fig. 6B–C). In baseline seropositive subjects, vaccination induced transient decreases in the percentage of Ad5-specific effector CD8^+^ T-cells (Fig. 6C, p<0.05) that corresponded with an increase in the percentage of Ad5-specific effector memory cells (CD27-CD45RO^+^; Fig. 6D, p<0.05). In baseline seronegative subjects the effector phenotype observed at baseline remained stable following vaccination with only a transient increase in the percentage of Ad5-specific memory cells observed at week 26 (Fig. 6E). In the total CD8^+^ T-cell pool, the effector phenotype dominated in both serogroups at baseline. Following vaccination the percentage of all effector CD8^+^ T-cell (Fig. 6F) in seropositive subjects decreased, coinciding with an increase in all central memory-like CD8^+^ T-cells (CD27^+^CD45RO^+^; Fig. 6H). In seronegative subjects only transient changes in total CD8^+^ phenotype occurred (Fig. 6F–H). Thus, while transient changes in memory phenotype were observed after vaccination, there were no sustained alterations in the memory phenotype of total or Ad5-specific CD8^+^ T-cells in either of the cohorts. These data suggest that Ad5-based vaccination does not induce global bystander CD8^+^ activation.

## Discussion

Recombinant Ad vectors are one of the primary vaccine platforms that are being tested for a wide range of human pathogens including HIV, malaria, and tuberculosis [6], [27], [28]. While these studies primarily focus on the generation of immune responses against the recombinant insert, an often-overlooked issue is the induction of vector specific immunity. Here we have examined Ad5-specific CD8^+^ T-cell responses in recipients of an Ad5 HIV-1 vaccine candidate.

To effectively measure the magnitude of Ad5-specific CD8^+^ T-cell responses against the entire Ad5 vector, we developed a novel assay for stimulating PBMCs overnight with a recombinant Ad5 vector. We have previously demonstrated that the Ad5 vector infects antigen presenting cells and is capable of stimulating Ad5-specific T-cell responses [17]. One limitation of these results is the possibility that we are detecting CD8^+^ T-cell responses against the rabies glycoprotein insert. This is extremely unlikely as less then 40,000 people, or approximately 0.01% of the US population receives the rabies vaccine each year and would have pre-existing immunity to antigens of rabies virus [29]. Antigen presentation of structural proteins of E1-deleted Ad vectors through the MHC class I presentation pathway may be achieved through either cross-presentation or more likely by residual transcription of late Ad gene products that occurs even with E1-deleted vectors [30]. This latter notion is further supported by our observation that cells transfected with E1-deleted vectors produce hexon transcripts albeit at substantially lower level than cells infected with a corresponding dose of wild-type virus. Considering that T cells respond to minute amounts of their cognate antigen we would assume that such levels that escape detection by conventional methods designed to detect protein, more than suffice to trigger stimulation of T cells. One could argue that the Ad vector transcripts were analyzed in CHO cells rather than in PBMCs or antigen-presenting cells as previous publications have shown that complementation of the E1 function is cell type specific [25], [26]. We would like to point out that our previous studies have shown that Ad vectors readily transduce antigen presenting cells [31] and furthermore that Ad vectors persist in a transcriptionally active form in lymphocytes, especially activated T cells, suggesting that such cells most likely provide factors that allow for the expression of structural proteins of Ad vectors [32].

We find that regardless of baseline Ad5 nAb serostatus, Ad5 vector administration results in a potent restimulation and expansion of pre-existing Ad5-specific CD8^+^ T-cells. This finding by itself is curious. One would have expected that E1-deleted Ad vectors only produce trace amounts of structural Ad proteins, as the transcription of late genes is under the control of a gene product of the deleted E1 domain. Nevertheless, as has been shown by others, CD8^+^ T cell responses to Ad vector particle proteins can be induced efficiently by cross-priming. This mechanism would circumvent the need for de novo synthesis of Ad structural proteins for induction or recall of Ad-specific CD8^+^ T cells [33].

At baseline, Ad5-specific CD8^+^ T-cells were detectable in 38 of 40 subjects, despite a seroprevalence in the US of up to only 50%. This magnitude of Ad5 -reactive CD8^+^ T-cells is consistent with smaller studies showing Ad5-specific CD8^+^ T-cell responses in greater than 80% of subjects [14], [15], [16]. The prevalence of Ad5-specific CD8^+^ T-cells is likely the result of cross-reactive CD8^+^ T-cell generated from infection with alternate serotypes. Many Ad proteins have highly conserved regions between various Ad serotypes, likely resulting in conservation of T cell epitopes and the generation of cross-serotype reactive Ad5-specific CD8^+^ T-cells. Although we were able to observe a high frequency of responders after stimulation with an Ad5 vector *in vitro*, it is unclear whether detected responses were induced by a natural infection with an Ad5 virus.

Although vaccination increased frequencies of Ad5-specific effector CD8^+^ T-cells in both serogroups, there were no significant changes either within or between the groups for the cell cycle marker Ki67. Though the PBMC sampling in this study was intensive, there was a four-week period between vaccination and PBMC collection. It is possible that expansion of Ad5-specific T-cells and expression of Ki67 occurred transiently during this period. Alternatively, Ki67^+^ Ad5-specific CD8^+^ T-cells may have trafficked out of the peripheral blood by this time.

Interestingly, we observed no changes in polyfunctionality of Ad5-specific CD8^+^ T-cells compared to baseline following vaccination in either serogroup. As we have previously observed [17], Ad5-specific CD8^+^ T-cells are continuously maintained in both a functionally and phenotypically effector-like state in most individuals, likely reflecting continued or intermittent exposure to Ad viruses. Ad5 vector administration did not alter this functional response, and instead further expanded it. This expansion could potentially reduce the effectiveness of Ad vaccine boosting with both homologous and heterologous Ad vectors by the direct elimination of vector-transduced cells.

## Supporting Information

Figure S1 Study Design. Frozen PBMCs from 40 subjects who had previously participated in a Merck Ad5 gag/pol/ned phase I trial (016) were utilized for this smaller basic immunology study. The study consisted of 25 unvaccinated subjects used for baseline comparison, five baseline seronegative subjects who received one dose 3×1010 vp Merck Ad4 gag/pol/nef at week 0 and were analyzed at weeks 0 and 4, five baseline seronegative subjects who received three dose 3×1010 vp Merck Ad4 gag/pol/nef at week 0, 4 and 26 and five baseline seropositive subjects who received three dose 3×1010 vp Merck Ad4 gag/pol/nef at week 0, 4 and 26. For subjects receiving three doses, PBMCs were analyzed from weeks 0, 4, 8, 18, 26, 30, 42, 52 and 78.
(0.06 MB TIF)
