# Supplementary figures and images for: Vaccination with Ad5 Vectors Expands Ad5-Specific CD8+ T Cells without Altering Memory Phenotype or Functionality
Source: PLoS One. 2010 Dec 22;5(12):e14385. doi: 10.1371/journal.pone.0014385 (PMC3008674; doi:10.1371/journal.pone.0014385)

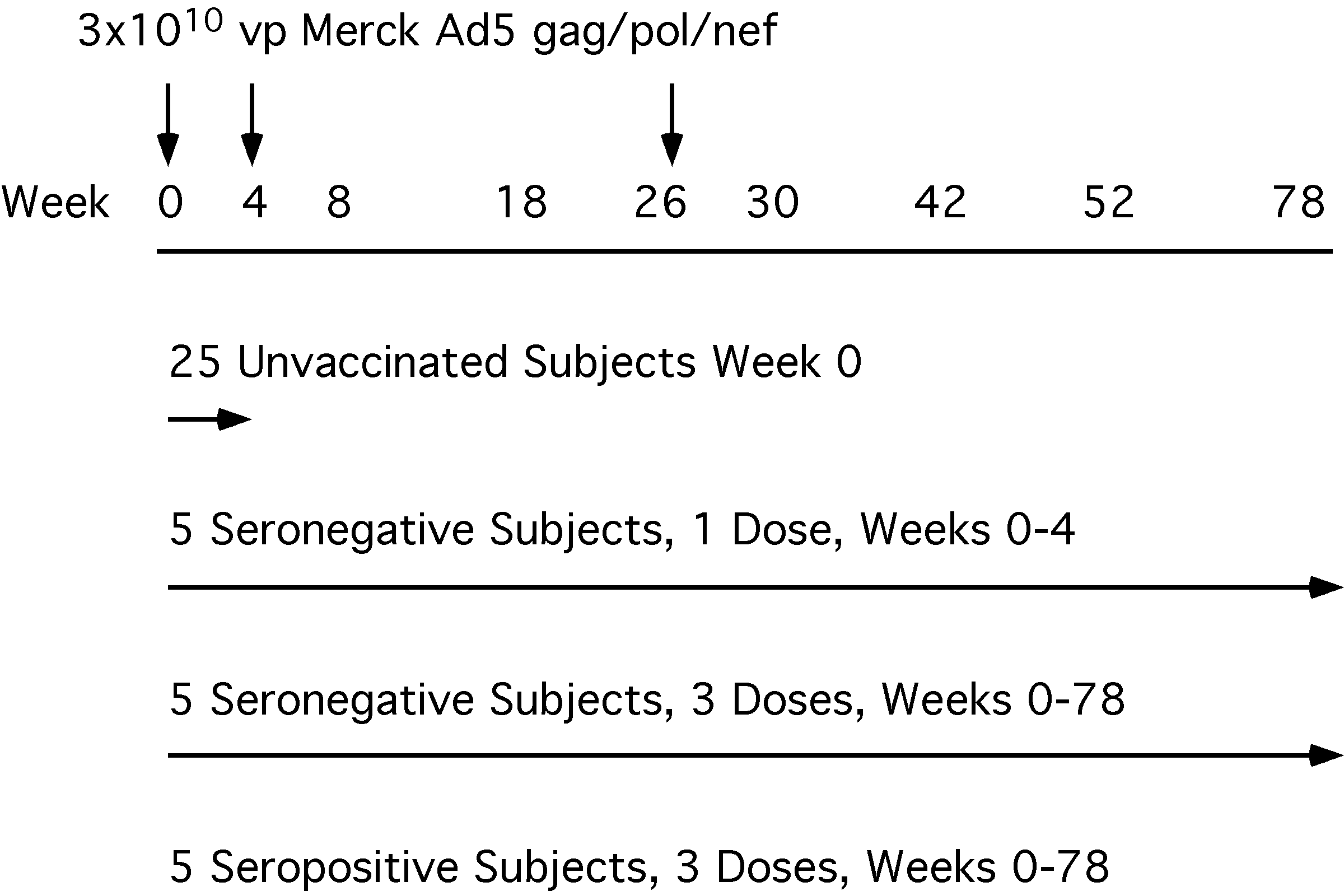

Supplement: Figure S1 — Study Design. Frozen PBMCs from 40 subjects who had previously participated in a Merck Ad5 gag/pol/ned phase I trial (016) were utilized for this smaller basic immunology study. The study consisted of 25 unvaccinated subjects used for baseline comparison, five baseline seronegative subjects who received one dose 3×10^10^ vp Merck Ad4 gag/pol/nef at week 0 and were analyzed at weeks 0 and 4, five baseline seronegative subjects who received three dose 3×10^10^ vp Merck Ad4 gag/pol/nef at week 0, 4 and 26 and five baseline seropositive subjects who received three dose 3×10^10^ vp Merck Ad4 gag/pol/nef at week 0, 4 and 26. For subjects receiving three doses, PBMCs were analyzed from weeks 0, 4, 8, 18, 26, 30, 42, 52 and 78. (0.06 MB TIF) [file pone.0014385.s001.tif]
